# The Influence of Blast Furnace Slag on Cement Concrete Road by Microstructure Characterization and Assessment of Physical-Mechanical Resistances at 150/480 Days

**DOI:** 10.3390/ma16093332

**Published:** 2023-04-24

**Authors:** Liliana Maria Nicula, Daniela Lucia Manea, Dorina Simedru, Oana Cadar, Anca Becze, Mihai Liviu Dragomir

**Affiliations:** 1 Faculty of Civil Engineering, Technical University of Cluj-Napoca, 28, Memorandumului Street, 400114 Cluj-Napoca, Romania; mihai.dragomir@cfdp.utcluj.ro; 2 Faculty of Construction, Cadastre and Architecture, University of Oradea, 4, B.S. Delavrancea Street, 410058 Oradea, Romania; 3 INCDO-INOE2000, Subsidiary Research Institute for Analytical Instrumentation Cluj-Napoca, 67 Donath Street, 400293 Cluj-Napoca, Romania; dorina.simedru@icia.ro (D.S.); oana.cadar@icia.ro (O.C.); anca.naghiu@icia.ro (A.B.)

**Keywords:** concrete shrinkage, mechanical resistances, carbonation, concrete microstructure, ground granulated blast furnace slag (GGBS), air cooled blast furnace slag (ACBFS), cement concrete roads

## Abstract

The results presented in this paper on the appropriateness of using of blast furnace slag (BFS) in the composition of roads make an original contribution to the development of sustainable materials with the aim to reduce the carbon footprint and the consumption of natural resources. The novelty of this work consists of determining the optimal percentage of BSF in road concrete, in order to: increase mechanical resistances, reduce contractions in the hardening process, and ensure increased corrosion resistances, even superior to classic cement-based mixtures. Thus, the physical-mechanical characteristics and the microstructure of some road concretes were studied in the laboratory for three different recipes. We kept the same amount of ground granulated blast furnace slag (GGBS) as a substitute for Portland cement, respectively three percentages of 20%, 40%, 60% air-cooled blast furnace slag (ACBFS) and crushed as sand substitute from now on called S54/20, S54/40, S54/60. Drying shrinkage, mechanical resistances, carbonation-induced corrosion, microstructure characterization of hardened concretes, and degree of crystallinity by SEM and XRD measurements were analyzed after a longer curing period of 150/480 days. The obtained results on the three BSF mixtures indicated a reduction of drying shrinkage and implicitly increased the tensile resistance by bending to 150 days well above the level of the blank composition. The degree of crystallinity and the content of the majority phases of the mineralogical compounds, albites, quartz, and tobermorite out of the three BSF samples justifies the increase in the compressive strengths at the age of 480 days in comparison with the test samples. Scanning electron microscope (SEM) and X-ray diffraction measurements showed the highest compactness and lowest portlandite crystal content for the S54/20 slag composite. Future research concerns are the realization of experimental sections in situ, the study of the influence of BFS on the elasticity module of road concrete, and the opportunity to use other green materials that can contribute to the reduction of the carbon footprint, keeping the physical and mechanical properties of road concrete at a high level.

## 1. Introduction

The construction industry has developed numerous measures to reduce the greenhouse gas emissions associated with cement production [1,2]. Moreover, the hydraulic cement manufacturing process is responsible for approximately 7–9% of global carbon dioxide emissions, on par with emissions from fuel combustion [3,4,5]. There are several materials resulting as byproducts or industrial waste that can be used as multi-component binders in cement mixtures to minimize the carbon footprint [3,6,7,8,9,10,11,12,13,14]. Using industrial byproducts such as GGBS from blast furnace iron ore extraction as a substitute for cement can reduce greenhouse gas (GHG) emissions by 47.5% [15,16]. The global environmental emission factor for producing of one ton of GGBS is 0.143 t CO_2_-e/ton, well below the value of 0.91 t CO_2_-e/ton for cement, which includes the cement transporter to the plants for the concrete mixture preparation [17]. Globally, slag production (GGBS) is almost 530 million tons, of which only 65% is absorbed by the construction industry [18,19]. Research has been carried out on the influence of GGBS on the performance of some types of concrete and mortar [3,20,21]. The research done in the study [22] shows that the use of an optimal combination of microsilica (MS) and GGBS can improve the resistance characteristics of concretes compared to the individual use of these additional cementitious materials. The experimental investigations within the paper [23] show that geopolymer concrete with GGBS as the main binder and substitution of slag powder with 20% micronized biomass silica (MBS) made from rice husk without Portland cement achieved optimal resistance and durability performance. The effects on the durability characteristics of waste glass-derived nanopowder (WGBNP) with the inclusion of fly ash (FA) and ground blast furnace slag (GBFS), evaluated on alkali-activated mortars (AAM), led to improved durability performances through reduced drying shrinkage and increased resistance to sulfuric acid, wear, and freeze-thaw cycles [24]. According to other research, the optimal dosage for replacing cement with GGBS is limited to max. 20%, because the resistances decreased significantly above this level compared to the reference concrete, due to low workability and increased porosity [15,25].

The impact of GGBS mixed with cement develops properties in fresh and hardened concrete, such as workability, reduced bleeding of fresh concrete, and hydration heat, increases long-term resistance, and increased resistance to corrosion, porosity, and low permeability [26,27,28,29]. The study [3] shows an increase in the workability of concrete up to a 40% substitution level with GGBS. Increased consistency is due to better particle dispersion (GGBS) [30]. More cement paste minimizes internal friction between concrete components by filling the micro-spaces in the concrete aggregate, resulting in more workable concrete [25]. Developing tensile strength, the main characteristic of road concrete, requires the careful establishment of design parameters for any structural element that requires crack control [31]. An important clue for determining the cracking resistance of concrete results from the evolution of shrinkage and the maximum level of shrinkage. The drier the air and the higher the temperature, the stronger the shrinkage [32]. Materials such as cement, water, and aggregates significantly influence concrete shrinkage. Cement leads to increased drying shrinkage by increasing dosage, tricalcium aluminate C3A component, gel component, alkali content of cement, deficiency, and excess of gypsum influence shrinkage curing [33]. The volume contraction of the cement paste represents approx. 1% of the absolute volume of dry cement [33,34]. Currently, the shrinkage has a value below 0.6 mm/m; this can be exceeded in concretes rich in binders [35]. Some authors appreciate the evolution of concrete shrinkage as follows: after ½ month 5% is recorded, after 3 months 60%, and after a year 75% of its maximum value [32]. The increase in the A/C ratio leads to an increase in concrete shrinkage, because the number of concrete pores also increases. Increasing the amount of aggregates reduces shrinkage due to their nature, rigidity, and granularity [36]. An increased concentration of CO_2_ and a humidity greater than 50% causes an increase in shrinkage to a value more than double. Carbon dioxide decalcifies and dehydrates hydrosilicates of the C_2_S type [35]. Increasing CO_2_ concentrations in the external environment also increases the carbonation rate for permeable concretes [37]. The carbonation phenomenon lowers the pH value from the typical values (12–13) to less than 9 in the pore solution and destroys the passivity of the reinforcing bars embedded in the concrete, triggering the corrosion process [38,39,40].

Another significant impact on the environment is the consumption of aggregates since they represent the largest share of the mass and volume of concrete. It is estimated that worldwide demand for construction aggregates is over 10 billion tons annually [41,42]. Much research explores the durability of concrete containing recycled aggregates (RAC) from building demolition [43,44], from road asphalt pavements (RAP) [45,46,47], quarry sand (QS) [48], or ecological mortars in which natural aggregates have been replaced by glass waste [49].

A sustainable source for substituting natural aggregates in road concrete is air-cooled crushed blast furnace slag (ACBFS) [50,51]. There are research studies related to the use of blast furnace slag as a substitute for natural aggregates in asphalt mixtures leading to these conclusions [52]. The Japanese Guide using blast furnace slag aggregates for concrete structures recommends using fine aggregates mixed with natural fine aggregates at a ratio between 20 and 60% [53]. In our country, since 2003, the characteristics of blast furnace slag aggregates used for concrete have been covered by the SR EN 12620 standard [54]. These byproducts from the steel industry can be used in road concrete mixes, but require proper evaluation to ensure that their properties do not adversely affect fresh and hardened concrete [55]. Applying reusable artificial materials and new technologies will positively impact on the environment by protecting non-renewable materials and reducing production costs [56,57].

The objective of this study is to continue investigations into the physical and mechanical characteristics of road concrete compositions with blast furnace slag made in previous works [58,59], to establish the optimal percentages of BSF to improve the quality of durability in concrete road production. It is known that cements containing blast furnace slag are characterized by a slower hydration rate, lower hydration heat, higher resistance after longer hardening periods, and greater resistance to chemical aggression [60]. The curing mechanism specific to concretes containing SCMs, such as GGBS, makes it possible to measure reference mechanical resistances after the age of 56 days [32]. Most concrete research with GGBS monitors curing times at 56 days [25] and 90 days [3] and fewer up to 360 days [61]. The relative humidity of the environment has a great influence on the size of the contraction. Thus, for a relative humidity of 100%, contraction decreases with age, while for a relative humidity of 50–70% (specific to road concretes that are kept in the air), the contraction can increase up to 20 years [32]. Variations in drying contraction influence the development over time of mechanical resistances (stretch and compression) and durability such as carbonation depth. This study aims to examine the influence of powdered blast furnace slag (GGBS) on the road concretes, analyzing the evolution of contraction and long-term resistance, 150/480 days. More than most previous research, road concrete compositions added fine aggregates (ACBFS) made of blast furnace slag. Consequently, tests were carried out on the samples made in the laboratory to evaluate some physical and mechanical properties (shrinkage on drying, tensile strength by bending at 150 days, and compressive strength at 480 days), durability (corrosion from carbonation at 150 and 480 days old), and microstructural properties (X-ray diffraction and SEM scanning electron microscopy at 480 days old). Mechanical resistance values obtained were reported at the reference age of 28 days.

## 2. Materials and Methods

### 2.1. Materials

As binder, CEM I 42.5R cement acquired from Holcim Romania was used according to SR EN 197-1 [62] and GGBS from local sources, Galati Steel Mill (in Romanian). The 28-day activity index of granulated slag, amounting to 0.95, was taken from the manufacturer’s tests. By grinding BSF to a size smaller than 63 µm, slag activation by grinding fineness was pursued [63,64,65]. The GGBS powder recorded after grinding a specific surface area of 3775 cm^2^/g lower than that of Portland cement of 4385 cm^2^/g. The GGBS characteristics were analyzed according to the system (CaO-SiO_2_-Al_2_O_3_-MgO), in compliance with SR EN 15167:1 [66], from the XRF spectral analysis by the manufacturer. The requirements of this standard require that the sum of the masses of CaO + MgO + SiO_2_ be greater than 2/3 and the mass ratio (CaO + MgO)/(SiO_2_) > 1. The values recorded in Table 1 show that these requirements are met.

The aggregate (ACBFS) crushed to a size of 0/4 mm was produced by the Galati Steel Mill. XRD measurements performed on the aggregates (ACBFS) in the paper [58] indicated the percentage of 100% crystalline phase, the danger of disaggregation being removed [35]. The fineness modulus (Mf), evaluated in Table 2, resulting as the sum of the total percentages retained on the site series, placed the aggregates (ACBFS) in the category of large-grained sands with values between 2.4 and 4.0 and fine natural aggregate (NA) in the category of sand with medium grains having Mf between 1.5 and 2.8, in compliance with SR EN 12620 [54]. The water absorption coefficient WA_24_ for natural fine aggregates and ACBFS brought to the condition SS with the saturated surface and to the condition SSD with the dry saturated surface recorded the values in Table 2.

The selection of natural aggregates and the granulometric curve of the total mixture was in accordance with the requirements of the national standard NE 014 [67]. River sand (0/4), crushed river gravel (4/8), and crushed quarry screening were used for sorting (8/16) and (16/25) acquired from local sources (Balastiera Beclănuț and Bologa quarry). When preparing the concrete, the superplasticizer additive Master-Glenium SKY 527, (SP MG-SKY 527) and the air trainer additive Master Air 9060 (MA 9060) were added, having characteristics in compliance with SR EN 934-2 [68]. The additives (Ad) used were acquired from the Master Builders Solutions Romania group. The water was taken from the supply system of the city of Cluj-Napoca, the characteristics being in compliance with SR EN 1008 [69].

### 2.2. Concrete Mixtures

Five mixtures were made, of which the first two compositions were made with conventional materials, with Portland cement, CEM I 42.5R, and natural aggregates. In three mixtures, GGBS and the ACBFS aggregates were added. A quantity of 54 kg/m^3^ of slag (GGBS) was used as an addition in the percentage of 15% of the mass of cement in the control composition S360, as a substitute in the percentage of 13% of the mass of cement in the control composition S414. The sand (NA) was substituted in percentages of 20%, 40%, and 60% with crushed aggregates (ACBFS) with a size of 0/4 mm.

The amounts of materials per m^3^ are summarized in Table 3, and the abbreviations for mixtures are symbolically noted as follows.

In the preparation of slag concretes, the added water content and additives were helped to achieve consistency within the range 20–40 mm, workability being an important feature of road concretes.

The design parameters of road concrete classified in the BcR 5.0 class followed the requirements set out in NE 014 [67], presented in Table 4, and the obtained results are presented in detail in the paper [59].

### 2.3. Methods

#### 2.3.1. The Drying Shrinkage of Road Concretes with Blast Furnace Slag

**Figure 1:**
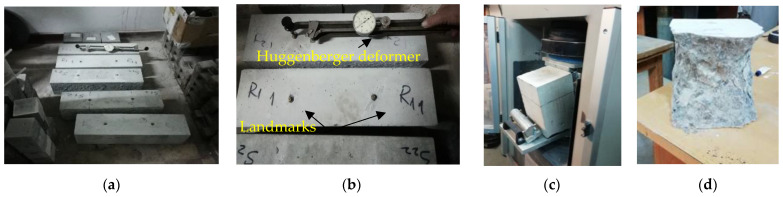
(**a**,**b**) Pictures of drying shrinkage measurement; (**c**) Images after the bending tensile test of the 150 × 150 × 600 mm prism; (**d**) Images after the compression test on the 150 mm cube.

The shrinkage of hardened concrete was measured in compliance with SR 2833 [70], with the help of the Huggenberger deformer, the landmarks being located at a distance of 250.50 mm. The shrinkage measurement was carried out on prisms with dimensions of 150 × 150 × 600 mm^3^. The samples were kept in water until the age of 7 days, then in air until the age of 150 days at a humidity of 65 ± 5% and a temperature of 20 ± 2 °C, as in Figure 1a,b. The initial reading was taken at 7 days, followed by further readings at 14, 28, 42, 56, 90, and 150 days. At each test age, the shrinkage was calculated as the arithmetic mean of the values obtained on three samples on three test tubes, applying (Equation (1)):

(1) $$\varepsilon_{\mathrm{ci}}=\frac{\delta_{0}-\delta_{i}}{l}\left(\frac{\mathrm{mm}}{m}\right),$$

In which:

ε_ci_—shrinkage of the hardened concrete, in mm/m;
δ_0_—initial reading at 7 days old (standard) with deformer, in mm;
δ_i_—reading at the age of i days, with deformer, in mm;
l—distance between landmarks, in mm.

#### 2.3.2. Tensile Strengths by Bending, Compression, and Carbonatation

The determination of mechanical resistances was carried out on three prism test tubes, with dimensions of 150 × 150 × 600 mm^3^, respectively on three cubes with a side of 150 mm for each composite. The prismatic samples for determining the bending tensile strength in compliance with SR EN 12390-5 [71] were the same as those monitored for drying shrinkage evaluation up to the age of 150 days, images in Figure 1c. The compressive strengths were determined in compliance with SR EN 12390-3 [72], after a longer curing period, at the age of 480 days, images Figure 1d. The evolution of the mechanical resistances was compared to those at the reference age of 28 days, found in the paper [59]. The determination of the resistance to carbonation at 150/480 days was performed on the freshly crushed faces of the three cubes and prisms remaining after the mechanical tests from each composite. According to the methodology of SR CR 12793 [73], they were sprayed with 1% phenolphthalein solution to measure the depth of the carbonation layer dk (mm).

#### 2.3.3. Characterization of the Microstructure of Road Concrete

The XRD patterns were recorded using a D8 Advance diffractometer (Bruker, Karlsruhe, Germany) with Ni-filtered CuK α1 radiation of λ = 1.54060 Å wavelength, operating at 40 kV and 40 mA, at room temperature. The degree of crystallinity was determined as the ratio between the area of diffraction peaks and the total area of diffraction peaks and halos.

SEM-EDX scanning electron microscope measurements were performed on small samples at the age of 480 days. The SEM-EDX analysis was performed at room temperature using a scanning electronic microscope (VEGAS 3 SBU, Tescan, Brno-Kohoutovice, Czech Republic) with a Quantax EDX XFlash (Bruker, Karlsruhe, Germany) detector. Samples of ~4 mm^2^ were mounted with carbon tape on an SEM stub. For each composite, XRD-SEM-EDX measurements were performed on a single sample.

## 3. Results

### 3.1. The Drying Shrinkage of Road Concretes with Blast Furnace Slag

In the same conservation conditions (7 days in the humid environment and the rest in the air with controlled humidity), Table 5 shows the values of the contraction upon curing (ε) up to the age of 150 days.

### 3.2. Tensile Strengths by Bending and Compression

Tensile flexural strengths at 150 days, compression at 480 days, standard deviation (SD), and coefficient of variation (CoV) of mechanical strengths are given in Table 6.

### 3.3. Corrosion Resistances from Carbonation

**Figure 2:**
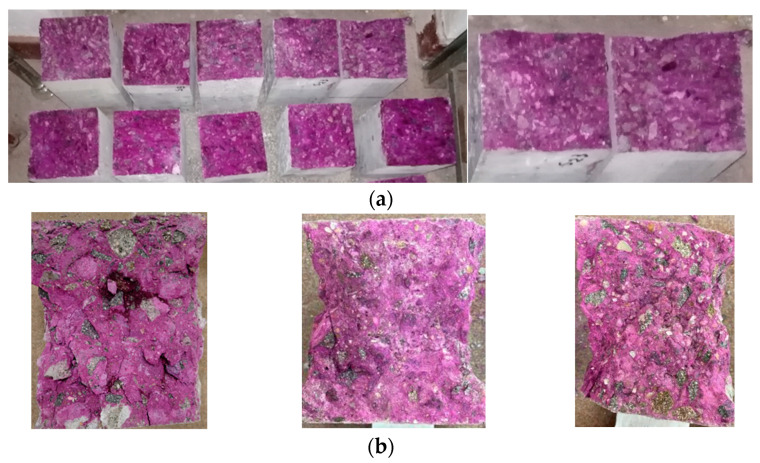
Samples after one hour of spraying with 1% phenolphthalein solution; (**a**) Fragments from prisms tested at the age of 150 days; (**b**) Fragments from cubes tested at the age of 480 days.

Figure 2a presents photo images of fragments from prisms tested at the age of 150 days. In Figure 2b, fragments from cubes were tested at the age of 480 days after one hour of spraying with phenolphthalein solution in a concentration of 1%.

### 3.4. Characterization of the Microstructure of Road Concrete

#### 3.4.1. X-ray Diffraction

**Figure 3:**
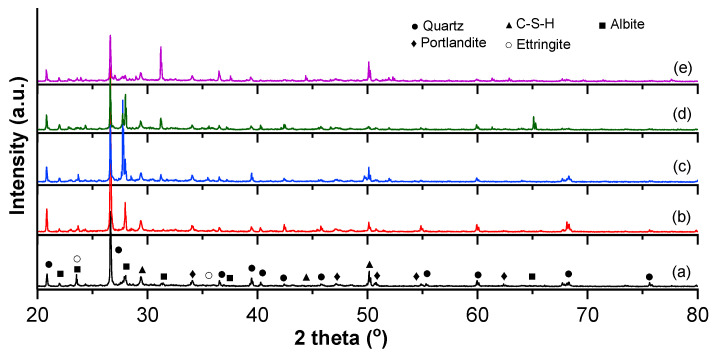
Diffraction patterns of the samples (**a**) S360; (**b**) S414; (**c**) S54/20; (**d**) S54/40; (**e**) S54/60.

The XRD diffraction patterns of the samples S360, S414, S54/20, S54/40, and S54/60 are presented in Figure 3.

XRD patterns showed the existence of quartz (SiO_2_), portlandite (Ca(OH)_2_), ettringite (Ca_6_Al_2_(SO_4_)_3_(OH)_12_·26H_2_O), calcium silicate hydrate (CaSiO_3_·H_2_O), and albite (NaAlS_i3_O_8_). The RIR (Reference Intensity Ratio) method was used for the quantitative phase analysis of the samples investigated at 480 days (Table 7).

#### 3.4.2. SEM-EDX Scanning Electron Microscopy Measurements

SEM investigations, at sizes from 20 µm to 500 µm, resulted in the surface topography for the compositions S360, S414, S54/20, S54/40 and S54/60, aged 480 days. Table 8 shows the pore size measured on the studied samples.

EDX was used to map the surface of the measured samples, the results for the concentration of the identified elements are presented in Table 9.

## 4. Discussion

### 4.1. The Drying Shrinkage of Road Concretes with Blast Furnace Slag

**Figure 4:**
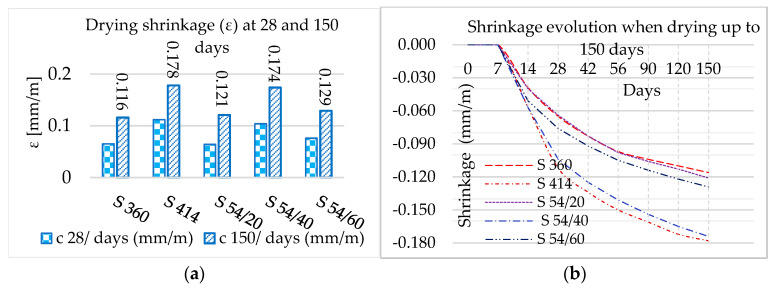
(**a**) Drying shrinkage at 28 and 150 days; (**b**) Evolution of drying shrinkage up to 150 days.

The highest shrinkage value was recorded for the S414 composite, 0.178 mm/m, which represents 63.33% of the maximum shrinkage value, respectively 0.283 mm/m, below the allowed value of 0.6 mm/m according to the principle presented in the paper [32,35], Figure 4a,b. The lowest shrinkage corresponds to the composite S360 in the amount of 0.116 mm/m. This result is justified by a reduced dosage of cement 360 kg/m^3^ compared to the dosage of 414 kg/m^3^ used for the rest of the composites. It is observed, for all composites, that the shrinkage value decreases with the decrease of the water content in the mixtures, recorded in Table 3.

Reducing the amount of cement by substitution with GGBS reduced shrinkage in slag composites. Moreover, from the pozzolanic reaction of GGBS with calcium hydroxide (CH), a lower drying shrinkage value resulted in samples with GGBS compared to the control mixture with the same binder dosage [12]. Increasing the amount of water while reducing the volume of aggregates in the S54/40 composite increased the shrinkage value compared to the S54/20 and S54/60 composites. The shrinkage mitigation of S54/20, S54/40, and S54/60 composites compared to S414 can be justified by the grinding fineness of GGBS with the specific surface (3775 cm^2^/g) lower than the specific surface of Portland cement (4385 cm^2^/g). Finer ground cements react more energetically with water and develop greater shrinkage after setting through stronger hydration and greater increase in the amount of gels [32].

The slag aggregates (ACBFS) also influenced the concrete shrinkage through grain size, porosity, and angularity greater than fine sand (NA) [74]. The ACBFS aggregates have larger grains than (NA) and higher porosity. After 24 h of immersion in water, the absorption coefficient was 10% higher in ACBFS aggregates compared to NA, the results in Table 2. Due to the crushing process, the angularity is higher in ACBFS aggregates than in NA. The larger grain size, shape, and porosity of ACBFS aggregates compared to NA lead to higher water absorption, in agreement with NA [75]. To avoid early shrinkage and reduction of freeze-thaw resistance, the slag aggregates must be brought to the SSD, a saturated state with the dry surface at the time of concrete preparation, as well as the use of a water-reducing admixture and an air-entraining admixture [76], conditions met in the laboratory for the compositions in this experiment.

### 4.2. Tensile Strengths by Bending and Compression

The tensile strength results show acceptable values between 3 and 6% for CoV and deviations between 0.19 and 0.35 MPa, of max. 6.06% for SD. For the compressive strengths, the coefficient of variation (CoV) of 4.65 MPa and the standard deviation (SD) of 0.6% with the highest value was recorded for the control composite S414. It can be appreciated that the dispersion of the results in Table 6 has a reasonable quality in a range from 1 to 6%, below the allowed limit of 15%, indicated in the paper [77].

**Figure 5:**
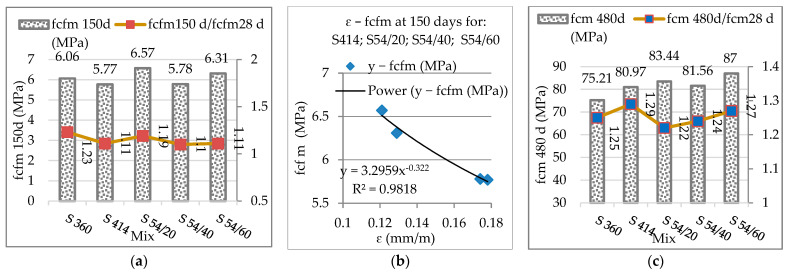
(**a**) The tensile strengths at 150 days and evolution coefficients from 28 to 150 days; (**b**) the relationship between contraction and tensile strength at 150 days; (**c**) compression strength at 480 days and evolution coefficients from 28 to 480 days.

Figure 5a presents the diagram of the tensile strengths at the age of 150 days and the evolution coefficients related to the reference resistances at 28 days. There is a more pronounced growth in the control composite S360 (1.23) and the composite with the furnace slag S54/20 (1.19) compared to the rest of the composites, in which the ratio was within the range 1.10–1.11. The decrease in tensile strength of the composite S414 (5.77 MPa) and S54/40 (5.78 MPa) has been largely influenced by the increase of shrinkage, caused by the superficial microcracking that develops especially at high cement dosages [32]. In Figure 5b, it is observed in the composites with high dosage of cement 414 kg/m^3^ a decrease in tensile strength with the increase of contraction, the function of the power between the two features registered a very good correlation coefficient (R^2^ = 0.98), confirming results also found in the paper [78]. Substitution (NA) with aggregate ACBFS led to increased tensile strength in the composite S54/20 (6.57 MPa) and S54/60 (6.31 MPa). The use of aggregate (ACBFS) increased adhesion to cement paste and tensile strength, because the asperities of the crushed aggregate surface are greater than those of NA [76].

Figure 5c presents the compression resistance diagram and evolution coefficients related to the reference resistances at 28 days. The highest compression resistance is observed for composite S54/60, closer for S54/20, and lower for S54/40, but the values registered are above the level of the control composites S360 and S414, at the age of 480 days. As expected, the evolution coefficients of mechanical resistances at the age of 480 are higher than those at 150 days compared to the reference attempts at 28 days. According to other studies, compression resistances have improved considerably after the age of 56 and 90 days of hardening for the concrete that contained GGBS. The improvement of compression resistances is attributed to the pozzolanic reaction of GGBS, which continues gradually compared to hydration (OPC) [79,80,81].

### 4.3. Corrosion Resistances in Carbonation

The photo images in Figure 2a,b show that the samples were not affected by the carbonation up to the age of 480 days, as the color of the indicator solution (red-purple) remained uniform throughout the surface of the cement stone, from the inner region to the outer edges of the samples. These results suggest that the diffusion of carbon dioxide has not occurred in the cement matrix, a phenomenon prevented by the compactness of the concrete cement stone [37,82].

### 4.4. Characterization of the Microstructure of Road Concrete

#### 4.4.1. X-ray Diffraction

**Figure 6:**
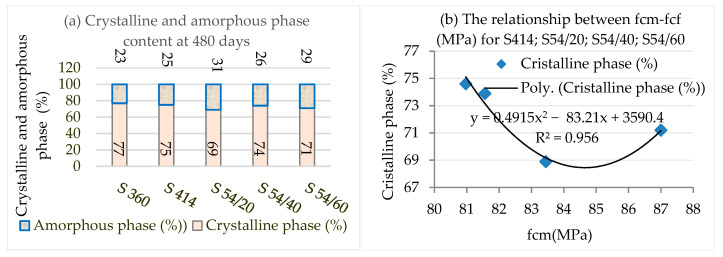
(**a**) The crystalline and amorphous phase content at 480 days of age: (**b**) Relationship between compressive strength and crystalline phase of S414, S54/20, S54/40, S54/60 samples at 480 days.

Albite (Ab) is a component of the plagioclase feldspar family, which is often present in the siliceous mineral aggregates that make up concrete [83] and in cement-based mortars containing recycled fine aggregates [84]. Ab is the major crystalline component for composites with blast furnace slag S54/20, S54/40, and S54/60, while for the control samples S360 and S414, quartz (SiO_2_) becomes the main crystalline phase. The C-S-H product, which influences the increase of mechanical resistance and impermeability [76,85], registers a high frequency for the S54/20 and S54/60 composites, a reduced frequency for the control (S360 and S414) compositions, and a low frequency for the S54/40 composite (Table 7). This evolution is consistent with the mechanical resistance results presented above. The CH product does not influence the mechanical resistances, but the addition of SCM in the cement paste can form additional C-S-H [86]. The ettringite (C-A-S-H) affects concrete structures due to the formation of secondary ettringite, which is expansive within the material [87]. After a longer curing period, the products (CH and C-A-S-H) display lower frequency. It is known that the presence of the amorphous phase leads to the development of a high pozzolanic activity [88]. The amorphous phase presented as the difference from the crystalline phase suggests the highest pozzolanic activity in the composites S54/20 and S54/60, followed by S54/40, all being above the level of control samples S414, a possible explanation being the addition of GGBS (Figure 6a). The slag (GGBS) reacts slowly in the presence of water but becomes reactive in the presence of calcium hydroxide (CH) in the pore solution of hydrated Portland cement [76]. For the same cement dosage, the compressive strengths increase with the decrease of the crystalline phase according to a second-order polynomial relationship, having a very good correlation coefficient (R^2^ = 0.96) (Figure 6b).

#### 4.4.2. Measurements with Electronic Microscopy with SEM-EDX Scanning

**Figure 7:**
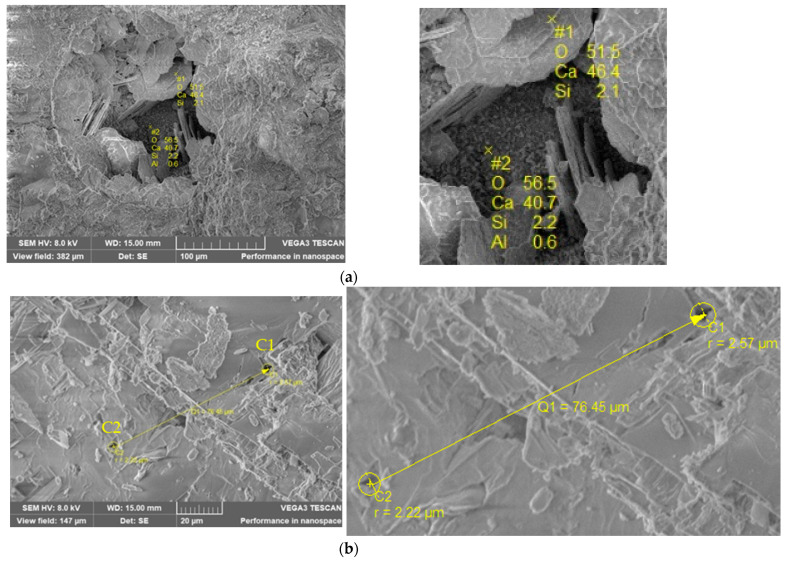

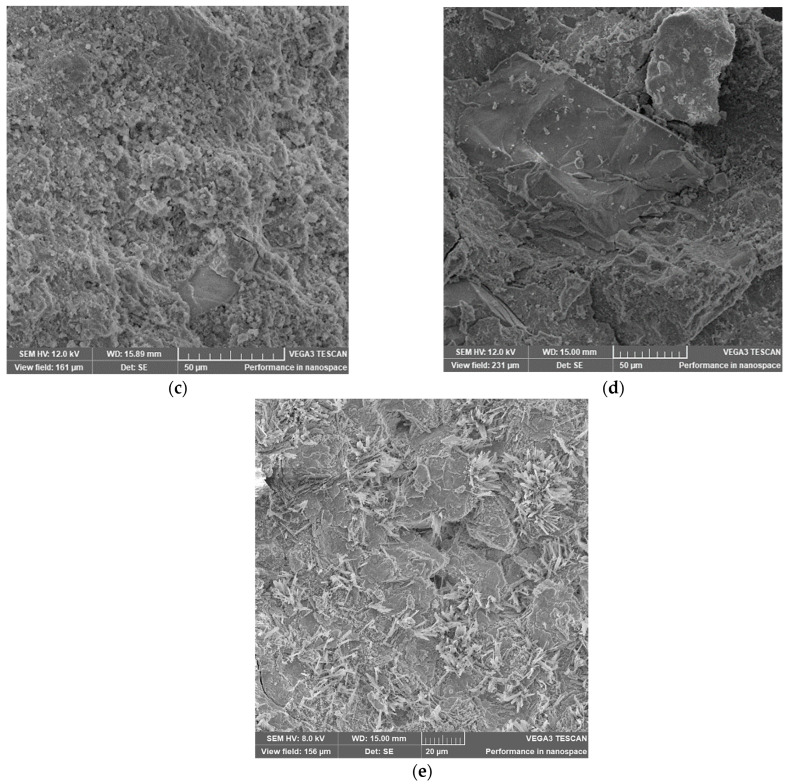
SEM images of (**a**) S360; (**b**) S414; (**c**) S54/20; (**d**) S54/40; (**e**) S54/60.

The topography, pore size, and morphology of S360, S414, S54/20, S54/40, and S54/60 samples at advanced age (480 days) were studied by SEM-EDX and are shown in Figure 7. For a more complex characterization of the pore structure, some authors use methods such as mercury intrusion porosimetry (MIP) and fractal dimensions [89,90,91]. Still, for this work, the SEM technique was used because it provides information about the surface structure and several information about the porosity of the sample up to the minimum size at which one can be identified. As can be observed, their surfaces are irregular and inhomogeneous with large pores characteristic of air holes (>several μm) [92]. The presence of several characteristic mineral phases can be observed. Figure 7a–e show higher magnification images of examined samples, giving more information about the structure on the surface of the sample. S360 (Figure 7a) has large pores filled with ettringite needles and calcium hydroxide platelets [93,94]. With increasing Portland cement content, S414, the presence of well-defined portlandite crystals [94] can be observed at high magnification. In the case of S54/20 and S54/40, the addition of slag leads to a decrease of portlandite crystals, on the surface of which only small crystals can be observed. For S54/60, the dendritic growth of CaCO_3_ [94] can be observed in addition to the calcium hydroxide platelets. SEM analysis is consistent with XRD patterns showing maximum peaks for portlandite at S360 and S54/60.

**Figure 8:**
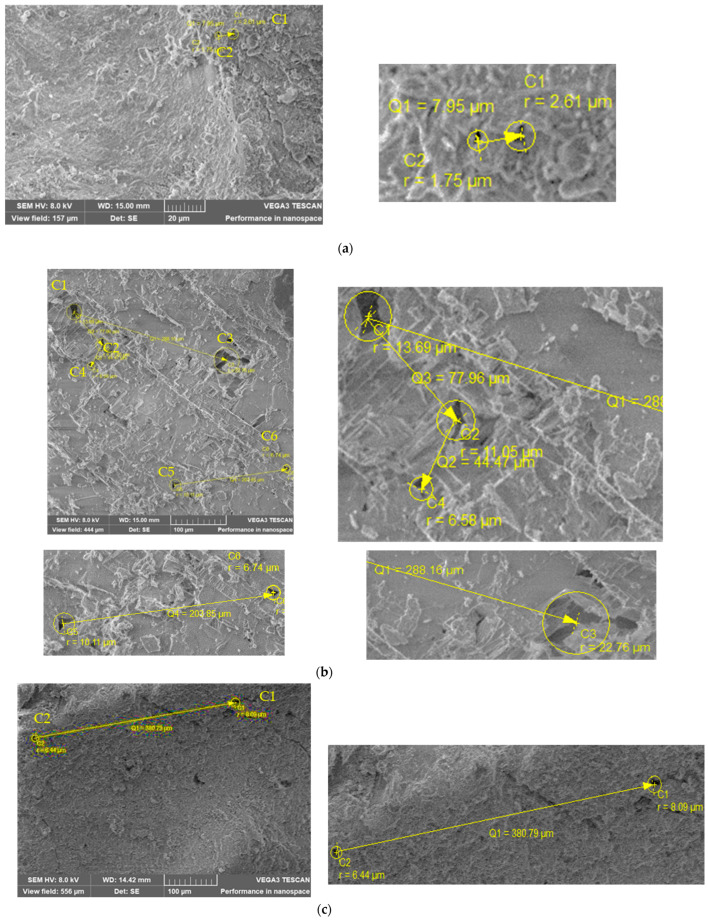

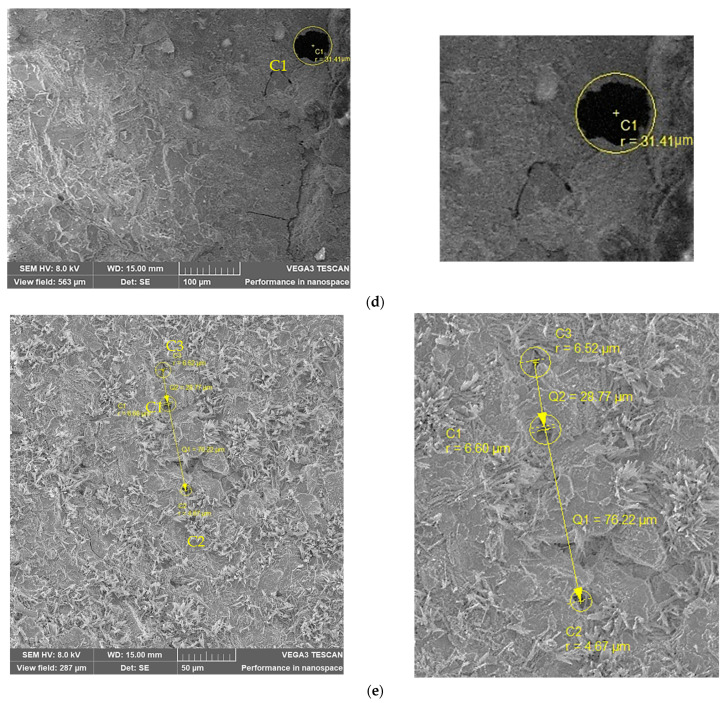
Pore structure of (**a**) S360; (**b**) S414; (**c**) S54/20; (**d**) S54/40; (**e**) S54/60 samples.

Knowing that the pore radius and spacing demonstrate the samples’ compactness, a comparison was made using the data in Table 8 and Figure 8a–e. The smaller pore radius (characteristic of air holes) is for samples S360 and S54/60 which show the highest content of portlandite crystals. The highest spacing between pores was obtained for S54/20, which shows the lowest content of portlandite crystals. The results indicate a correlation between the samples’ compactness and the portlandite crystals’ content. Portlandite being the most soluble constituent, it is the easiest to wash with water, it affects the cement stone through corrosion. The presence of a higher portlandite content in S360 and S54/60 results in smaller pores located at smaller a distance between them and thus a higher pore density. A lower quantity of portlandite content is observed in S54/20 which has higher pores at higher distance between them.

**Figure 9:**
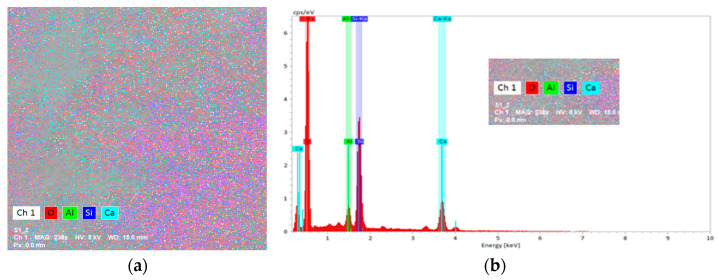

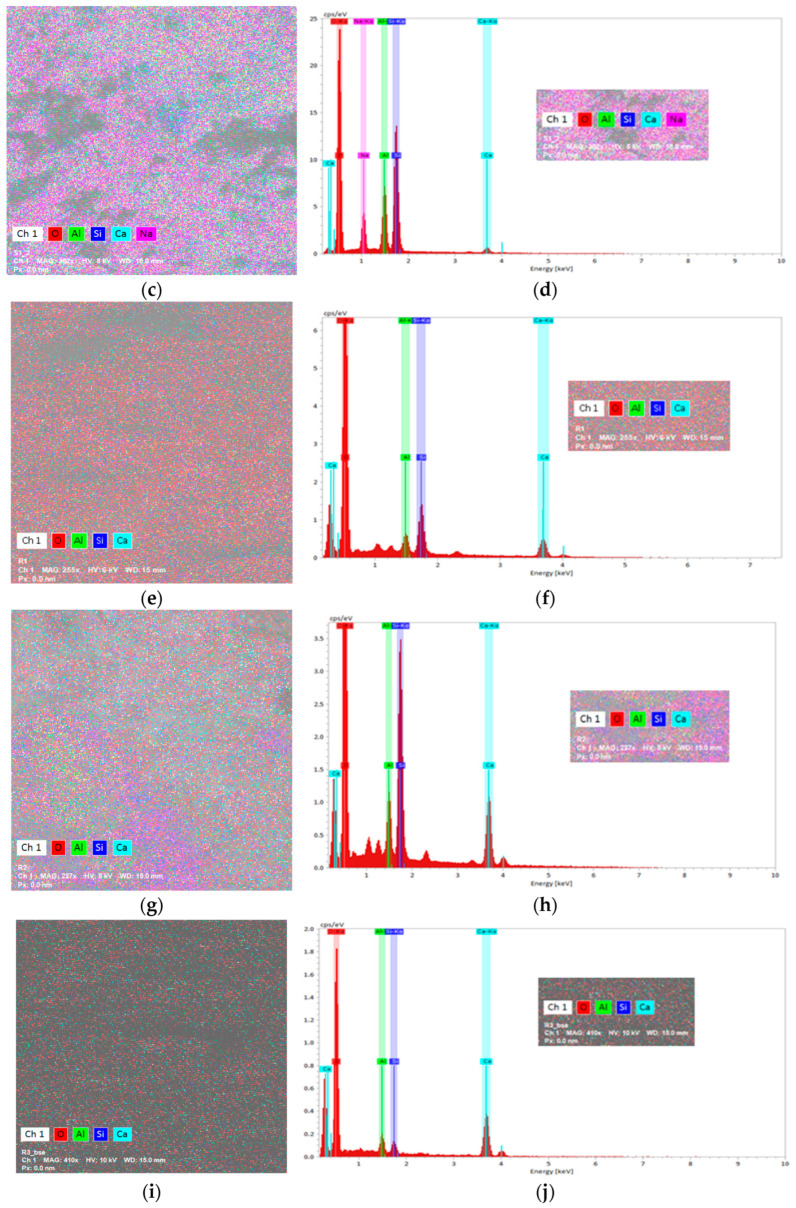
Surface mapping and EDX spectrum of (**a**,**b**) S360; (**c**,**d**) S414; (**e**,**f**) S54/20; (**g**,**h**) S54/40; (**i**,**j**) S54/60 samples at high magnification.

Figure 9a–j show the map surface and spectra of S360, S414, S54/20, S54/40, and S54/60 samples and the results obtained are presented in Table 9.

For all samples except S414, the predominant element besides oxygen is calcium. The results obtained for S414 indicate an inhomogeneity of the sample, with areas with many portlandite crystals and areas with small amounts of calcium, with the formation of silicon structures.

The ratio Ca/Si shows different variation:

- for S360 and S414, the values are below a value of 0.66 [95,96]. This indicates the formation of other silicate phases in the samples.
- for S54/20 and S54/40, the values are in the interval of values indicating the presence of different forms of C-S-H in the samples [95,96];
- for S54/60, the value of 12.86 indicates the presence of a majority of calcium-based structures.

## 5. Conclusions

In this paper, the performance of road concretes with GGBS and ACBFS were evaluated in the long term up to the age of 150 and 480 days. The results obtained from the evaluation of the contraction and the tensile strength at the age of 150 days, of the compression, carbonation, and microstructure characterization by the degree of crystallinity and the mineralogical composition at the age of 480 days lead to the following conclusions.

Up to the age of 480 days, the road concrete composites were not affected by carbonation corrosion.

XRD-SEM-EDX analysis suggests the lowest content of portlandite crystals for the S54/20 composite.

Very good compactness and low portlandite content result in the best corrosion resistance for the S54/20 blend.

The compressive strengths recorded the highest values for the compositions S54/20, S54/40, and S54/60.

The reaction products of the tobermorite group (C-S-H) as well as albite and quartz content were the main phases for the S54/20 and S54/60 composites with obtained the best mechanical strengths.

The reduction of the water-to-binding ratio as well as the grinding fineness of the GGBS powder diminishes the contractions in the slag mixtures compared to the control samples.

The reduction of hardening contractions positively influenced the tensile strength by bending to the composites S54/20 and S54/60 compared to the control composite S414.

Using crushed aggregate (ACBFS) with higher asperities than sand led to increased tensile strengths in S54/20 and S54/60 composites due to higher adhesion to the cement paste.

For the S54/20 and S360 composites, due to the decrease of contractions in the hardening process, there were the largest increases in bending tensile strengths at 150 days compared to 28 days.

For the same dosage of binder, the value of shrinkage during hardening decreased by 32.02% in the S54/20 composite and by 27.53% in the S54/60 composite compared to the control mixture S414 at the age of 150 days.

For the control composites, the main phase was quartz, while for the S54/40 composite it was albite. The addition of GGBS indicated a change in the morphology of the cement paste, favoring the formation of hydration product (C-S-H) for S54/20, S54/40, and calcium-based structures for S54/60.

The results presented in this paper contribute favorably to the opportunity and necessity of using road concrete containing blast furnace slag.

Future research should focus on the realizing of experimental concrete sectors with blast furnace slag subject to monitoring under the combined effect of temperature variations, traffic and chemical actions.

Last but not least, it is necessary to study the variation of the elasticity module in the case of slag road concretes as well as the opportunity to use other green materials that can reduce the carbon footprint, while maintaining the physical and mechanical properties at a high level.

## Figures and Tables

**Table 1:** Oxidative analysis of granulated slag (GGBS).

GGBS	SiO_2_	Al_2_O_3_	MnO	MgO	CaO	Fe_2_O_3_	Na_2_O	K_2_O
(%)	36.70	9.50	0.23	8.70	42.00	0.55	0.28	0.53

**Table 2:** Fineness modulus (Mf) and water absorption coefficient (WA_24_) of aggregates of size 0/4 mm [59].

Aggregate Mixture	Mf	WA_24_ (SS)	WA_24_ (SSD)
100% (NA)	2.72	20% (water saturated)	2% (after 4 days air cooled)
100% (ACBFS)	3.15	30% (water saturated)	2% (after 8 days air cooled)

**Table 3:** Quantities of materials per m^3^.

Mixtures (kg/m^3^)	S360	S414	S54/20	S54/40	S54/60
Cement	360	414	360	360	360
(GGBS)	-	-	54	54	54
Total binder (l)	360	414	414	414	414
Water (w)	166.47	174.39	172.91	181.23	167.64
w/l	0.46	0.42	0.42	0.44	0.41
(NA_0/4 mm)	607	594	478	355	240
(ACBFS_0/4 mm)	-	-	119	237	359
(CA_4/25 mm)	1290	1261	1269	1256	1275
(SP MG-SKY 527)	3.60	4.14	4.39	4.55	4.97
(MA 9060),	1.80	2.07	2.07	2.07	2.07

**Table 4:** The design parameters of road concrete composites in compliance with NE 014.

Cement Dosage	(w/l)	Consistency	Occluded Air Content	fcm 28 Days	fcfm 28 Days
min. 360 kg/m^3^	max. 0.45	(30 ± 10) mm	(3.5 ± 0.5)%	min. 50 MPa	min. 5.5 MPa

**Table 5:** Curing shrinkage of road concretes up to the age of 480 days.

Curing Shrinkage	S 360	S 414	S 54/20	S 54/40	S 54/60
ε (mm/m)—14 days	0.039	0.057	0.040	0.057	0.051
ε (mm/m)—28 days	0.065	0.112	0.064	0.104	0.076
ε (mm/m)—42 days	0.083	0.134	0.083	0.125	0.092
ε (mm/m)—56 days	0.097	0.150	0.098	0.141	0.105
ε (mm/m)—90 days	0.104	0.161	0.106	0.154	0.114
ε (mm/m)—120 days	0.110	0.172	0.113	0.165	0.122
ε (mm/m)—150 days	0.116	0.178	0.121	0.174	0.129

**Table 6:** Tensile flexural strengths at 150 days, compression at 480 days, standard deviation (SD), and coefficient of variation (CoV) of mechanical strengths.

Mixture	S 360	S 414	S 54/20	S 54/40	S 54/60
f_cfm_ 150 days (MPa)	6.06	5.77	6.57	5.78	6.31
SD-f_cfm_ (MPa)	0.33	0.35	0.19	0.28	0.29
CoV-f_cfm_ (%)	0.05	0.06	0.03	0.05	0.05
f_cm_ 480 days (MPa)	72.5	80.97	83.44	81.56	87.00
SD-f_cm_ (MPa)	2.94	4.65	4.27	1.10	4.48
CoV-f_cm_ (%)	0.04	0.06	0.05	0.01	0.05

**Table 7:** Quantitative analysis results obtained by the RIR method (%) of the samples investigated at 480 days.

Sample	S 360	S 414	S 54/20	S 54/40	S 54/60
Degree of crystallinity (%)	77	75	69	74	71
Amorphous phase (%)	23	25	31	26	29
Albite (Ab)	++	++	+++	+++	+++
Quartz	+++	+++	+++	++	+++
Tobermorites (C-S-H)	++	++	+++	+	+++
Portlandite (CH)	+	+	+	+	+
Ettringite (C-A-S-H)	+	+	+	+	+

+++ major phase (>20%), ++ minor phase (5–10%), + phases in traces (<5%).

**Table 8:** Pore size was measured on the studied samples from the compositions S360, S414, S54/20, S54/40, and S54/60.

Sample	Pore Identification Code	Pore Radius (μm)	Pore Diameter (μm)	Distance Identification Code Qi (Ci-Ci+n)	Distance (μm)
S360	C1	2.61	5.22	Q1 (C1–C2)	7.95
	C2	1.75	3.51		
S414	C1	13.69	23.79		
	C2	11.05	22.10	Q1 (C1–C3)	288.16
	C3	22.76	45.51	Q2 (C2–C4)	44.47
	C4	6.58	13.17	Q3 (C1–C2)	77.69
	C5	10.11	20.21	Q4 (C5–C6)	203.85
	C6	6.74	13.48		
S54/20	C1	8.09	16.19	Q1 (C1–C2)	380.79
	C2	6.44	12.89		
S54/40	C1	31.41	62.83	-	-
S54/60	C1	6.60	13.20	Q1 (C1–C2)	76.22
	C2	4.67	9.34	Q2 (C1–C3)	28.77
	C3	6.52	13.05		

**Table 9:** Element concentrations (%) in S360, S414, S54/20, S54/40, and S54/60 obtained by mapping the sample surface.

Mixture	O	Ca	Si	Al	Ca/Si
S360	53.30	22.37	21.92	2.41	0.42
S414	50.83	4.45	30.44	9.07	0.15
S54/20	54.18	28.54	14.54	2.74	1.96
S54/40	55.57	21.68	19.31	3.44	1.12
S54/60	68.79	27.02	2.10	2.10	12.86

## Data Availability

All the required data that support the finding are presented in the manuscript.
